# SDC-Net: End-to-End Multitask Self-Driving Car Camera Cocoon IoT-Based System

**DOI:** 10.3390/s22239108

**Published:** 2022-11-24

**Authors:** Mohammed Abdou, Hanan Ahmed Kamal

**Affiliations:** 1 Valeo Egypt, Cairo 12577, Egypt; 2 Department of Electronics and Communications Engineering, Faculty of Engineering, Cairo University, Giza 12613, Egypt

**Keywords:** autonomous driving, deep learning, computer vision, multitask learning, crash avoidance, path planning, automatic emergency braking, camera-cocoon, IoT, system

## Abstract

Currently, deep learning and IoT collaboration is heavily invading automotive applications especially in autonomous driving throughout successful assistance functionalities. Crash avoidance, path planning, and automatic emergency braking are essential functionalities for autonomous driving. Trigger-action-based IoT platforms are widely used due to its simplicity and ability of doing receptive tasks accurately. In this work, we propose SDC-Net system: an end-to-end deep learning IoT hybrid system in which a multitask neural network is trained based on different input representations from a camera-cocoon setup installed in CARLA simulator. We build our benchmark dataset covering different scenarios and corner cases that the vehicle may expose in order to navigate safely and robustly while testing. The proposed system aims to output relevant control actions for crash avoidance, path planning and automatic emergency braking. Multitask learning with a bird’s eye view input representation outperforms the nearest representation in precision, recall, f1-score, accuracy, and average MSE by more than 11.62%, 9.43%, 10.53%, 6%, and 25.84%, respectively.

## 1. Introduction

Recently, deep learning and computer vision are invading the automotive field. Sophisticated features have become essential in order to achieve a self-driving car, including: lane keeping assist (LKA) [1], path planning [2], adaptive cruise control (ACC) [3], automatic emergency braking (AEB) [4], traffic jam assist (TJA) [5], and crash avoidance (CA) [6]. AEB aims to force a brake control especially in case of an object in front of the ego-vehicle, LKA aims to control the ego-vehicle steering angle within the same lane with at constant speed. ACC aims to control the ego-vehicle’s speed while keeping a safe distance away from the front vehicle. TJA aims to continuously measure the surrounding vehicles’ speed especially when ACC is activated; the car automatically follows the vehicle in front, accelerates, and brakes all by itself, at all speeds below 60 kmph. CA aims to detect the possibility of the occurrence of crashes and try to avoid them by taking corrective control actions. Path planning aims to take the relevant control steering and acceleration to maneuver and move the vehicle from the current lane to the next lane. All of the previously mentioned functionalities are not fully-integrated in all different ways and levels.

All of the previous actions are completed based on having a highly accurate level of environmental perception that depends on sensor fusion [7]. Sensor fusion is the ability to gather inputs from multiple sensors lidars, cameras, and radars in order to precept the environment around the vehicle. The outcome from this perception is robust and accurate because it combines the strength points of these different sensors. Each sensor has some strengths and weaknesses. For example, radar is often used in estimating distance and speed even in bad weather conditions [8]; however, radar fails in detecting traffic road signs. The use of a camera is common in these situations: classifying and detecting traffic signs, vehicles, pedestrians, etc.; however, cameras fail in the case of darkness, dirt, and sunny weather, so lidars are helpful in these situations for estimating distance from other objects.

Currently, crash avoidance, path planning [2] and automatic emergency braking functionalities are considered as the most important features in self-driving cars. These functionalities are not fully-integrated in all different ways and levels. For example, if there is an accident, the currently implemented solutions perform many actions such as:

- Warn the driver that there is an accident in front of them. Then, the driver will have the ability to take a reasonable corrective action. This is called the forward collision warning (FCW) functionality;
- Apply automatic emergency braking (AEB) functionality; however, this functionality is specific to low speeds.

The internet of things (IoT) allows devices to be connected wirelessly to a cloud system. The automotive industry has used IoT to cover difficult scenarios that self-driving cars may expose. Recently, self-driving cars have been coupled with an IoT-based technology system that shares huge amounts of information including traffic, navigation, roads, behaviors, scenarios, and more. Self-driving cars’ computer systems benefit from this information by doing extensive analyses to help vehicles move autonomously. With the advent of new telecommunication technologies such as 5G-networks [9] and vehicle-to-vehicle (V2V) communications [10], cooperative perception [11,12] is becoming a promising paradigm that enables sensor information to be shared between vehicles and roadside devices in real-time. The shared information can then augment the field of view of individual vehicles and convey the intentions and path plans of nearby vehicles, thus offering the potential to improve driving safety, particularly in accident-prone scenarios. Digital automation platforms are considered to be IoT platforms that are extremely useful services that enable users to connect applications and automate workflows. The software is currently directed towards specialized and centralized cloud applications. IoT [13] automation platforms are noncoding programming languagse that provides some APIs which can be automatically called or accessed. IoT platforms are a trigger-action programming method that facilitate the integration of many devices using a fascinating user interface and are capable of making some types of decisions.

In this paper, we introduce an SDC-Net (self-driving car) system which is an end-to-end multitask deep convolutional neural network. SDC-Net is designed to allow vehicles to navigate safely while performing tasks such as crash avoidance, path planning, and automatic emergency braking. In order to perform these tasks, we also created a dataset that serves our application using a CARLA simulator. A camera cocoon setup with views 360${}^{\circ }$ around the ego-vehicle—to have improved perception of the environment—was configured to capture images from front, left, right, and rear positions. Traditional path planning was implemented and integrated with the CARLA simulator using model predictive control (MPC) in order to control vehicle’s actions: steering, throttle, and braking with the help of the provided waypoints generated from the predefined map information from the CARLA simulator. Different scenarios and corner cases for path planning, crash avoidance, and automatic braking are covered in our dataset to navigate safely within CARLA towns. Our collected dataset (i.e., cocoon cameras with the corresponding control actions) is saved in order to retrieve the data easily while training or testing the multitask network. The SDC-Net system is also considered as an IoT-based solution that aims to enrich other connected vehicles with relevant information especially in case of accidents or crashes. Extensive experiments were conducted based on two combined factors: different input representations (front image, panorama, and bird’s eye view) and single/multi output head (crash avoidance only, path planning and AEB only, or both).

The remainder of the paper is distributed as follows: Section 2 provides a literature review on the self-driving vehicle functionalities, sensor fusion and different digital automation IoT platforms. Section 3 describes system architecture, neural networks models using different input representations, and the best automation IoT platform used. Furthermore, a literature review on open-source datasets and the dataset setup are described in Section 4. Moreover, experimental results are shown in Section 5. Lastly, a summarized conclusion is in Section 6.

## 2. Literature Review

Artificial intelligence (AI) is becoming more commonly used in many applications especially autonomous driving (AD) ones such as augmented reality (AR) [14], automatic emergency braking (AEB) [15], lane keeping assist (LKA) [16], active cruise control (ACC) [17], and crash avoidance (CA) [18]. Forward collision warning (FCW) [19] and automatic emergency braking (AEB) are considered as the initial trials to integrate crash avoidance functionality. In FCW, the vehicle is able to only warn the driver that there is an object in front of you, so the driver should take corrective actions. However, in AEB the vehicle starts taking action by braking when the vehicle approaches an object that is in front of the vehicle. Fully autonomous driving was forbidden due to the presence of some restricted laws that prevent autonomous cars because of the fear that accidents could occur. However, researchers have recently worked to create a research field with the aim of achieving a functioning self-driving car.

Path planning [20] is essential for autonomous driving cars because the vehicle requires full knowledge of the surrounding environment in order to take relevant actions. Path planning can be achieved using traditional methods or a deep learning approach:

- Perception [21], which is responsible for perceiving the surrounding environment;
- Trajectory prediction [22,23] for the surrounding objects;
- Trajectory planning [24], which computes the trajectory based on the perception and prediction for the environment;
- Control [25,26], which is responsible for taking proper actions depending on all the information that is gathered by the previous block.

Fusion of multiple sensors such as cameras, lidars, and radars which are widely used in self-driving vehicles, ensures accurate detection and robust perception even during bad weather conditions that may lead to a failure of some of the sensors. Recent related studies have developed vehicle detection methods by fusing lidar and radar signals together. STMVDNet [27] presents a teacher–student multimodal mutual learning framework in addition to a sensor noise model that can be used in data augmentation for both of these sensors.

Sensors are fundamental to conduct the perception task for self-driving cars that are exploring their surrounding environment. Fusing multiple sensor readings together can ensure a feasible autonomous vehicle. Wang et al. conducted a survey [28] discussing the different strategies of multisensor fusion in automated driving in recent years, including radars, lidars, cameras, ultrasonic sensors, and V2X. De Jong et al. conducted a review [29] evaluating the capabilities and the performance of the most commonly used sensors in autonomous vehicles, focusing on a large selection of vision cameras, lidar, and radar sensors with the many different conditions that sensors may experience. Their survey also presented an overview of the three primary categories of sensor calibration and reviewed existing open-source calibration packages for multisensor calibration and their compatibility with commercial sensors.

It is agreed that vehicle-infrastructure cooperation is required to achieve Level-5 full driving automation. Unfortunately, still, there is NO available real dataset from real scenarios available for researchers to work on vehicle-infrastructure cooperation-related problems. DAIR-V2X Dataset [30] is released to accelerate computer vision research and innovation for Vehicle-Infrastructure Cooperative Autonomous Driving (VICAD). DAIR-V2X Dataset is the first large-scale, multimodality, multiview dataset from real scenarios for VICAD, it comprises 70 K Lidar and Camera frames with 3D annotations. It is released for the problem of collaboratively locating and identifying 3D objects using sensory inputs from both vehicle and infrastructure, in addition to solving traditional 3D object detection problems taking into consideration the temporal asynchronous problem between vehicle and infrastructure sensors and the data transmission cost between them.

CoBEVT [31] is a multiagent, multicamera perception framework that can cooperatively generate BEV map predictions. It is a transformer-based architecture with a fused axial attention module (FAX). It aims to sparsely capture local and global spatial interactions across views and agents. It achieved the state-of-the-art performance for BEV semantic segmentation when tested on a OPV2V [32], which is a large-scale V2V perception dataset collected to facilitate multivehicle cooperation in CARLA [33] and OpenCDA [34], which is a generalized framework and tool for developing and testing cooperative driving automation (CDA) systems in a simulation. OPV2V employs a simple agent-wise single-head attention to fuse all features together.

DiscoGraph [35] is a novel distilled collaboration graph that aims to model trainable, pose-aware, and adaptive collaboration among agents that utilize IoT to share lidar information for better scene understanding. A teacher–student framework is used to train DiscoGraph via knowledge distillation [36], in which the teacher model employs an early collaboration with holistic-view inputs; the student model is based on intermediate collaboration with single-view inputs. Multiple agents with the shared DiscoNet could collaboratively approach the performance of a hypothetical teacher model with a holistic view. DiscoGraph was tested on V2X-Sim [37], which is a large-scale multiagent 3D object detection dataset for autonomous driving scenarios based on CARLA and the SUMO cosimulation platform [33]. It achieves better performance–bandwidth trade-off and lower communication latency than the state-of-the-art intermediate collaboration methods. V2X-Sim is a comprehensive simulated multiagent perception dataset for V2X-aided autonomous driving that provides multiagent sensor recordings from the roadside unit (RSU) and multiple vehicles that enable collaborative perception, multimodality sensor streams that facilitate multimodality perception, and diverse ground truths that support various perception tasks.

V2VNet [10] proposes to circulate the intermediate features extracted from 3D backbones (intermediate fusion), then utilize a spatial-aware graph neural network for multiagent feature aggregation. V2VNet is a vehicle-to-vehicle (V2V) communication setting where each vehicle can broadcast and receive information to/from nearby vehicles (within a 70 m radius). It also utilizes a spatially aware graph neural network (GNN) to aggregate the information received from all the nearby self-driving cars, which allows it to intelligently combine information from different points in time and viewpoints. It was also tested on V2X-Sim. COOPERNAUT [38] is an end-to-end learning model that uses cross-vehicle perception for vision-based cooperative driving. It encodes lidar information into compact point-based representations that can be transmitted as messages between vehicles via realistic wireless channels. The authors also developed a CARLA-based simulation framework AUTOCASTSIM with very challenging accident-prone scenarios.

Many research studies show the capabilities of digital automation IoT platforms for use in different IoT applications. In 2021 Mohammed Abdou et al. [39] conducted a comparative study between the five most famous digital automation platforms: Zapier [40], IFTTT [41], Integromat [42], Parapola [43], and Microsoft power automate [44]. The comparative study was based on different comparison metrics related to integrability, accessibility, and integrability. Valeo proposed an automotive standardization platform called digital gate system [45] that aims to connect a vehicle’s network (including CAN, Flexray, Ethernet, etc.) to cloud computation platforms as well as to the automation platforms and vice versa. Moreover, the digital gate system provides the capability of creating proof of concepts (POCs) and prototypes for automotive developers. Furthermore, it facilitates and accelerates the development cycle. Another comparative study was carried by Amir Rahmati et al. [46] differentiating between the capabilities of Zapier and IFTTT comparing the available functions, distribution of channels, and the common functions they share. Additionally, Amir Rahmati et al. analyzed the growth of these frameworks and discuss future research opportunities in this domain. In 2020, Lucas Carlberg and Olivia Shamon’s Bachelor thesis [47] researched the most optimal cloud integration platform, which allowed them to automate simple repetitive tasks.

## 3. System Methodology

**Figure 1:**
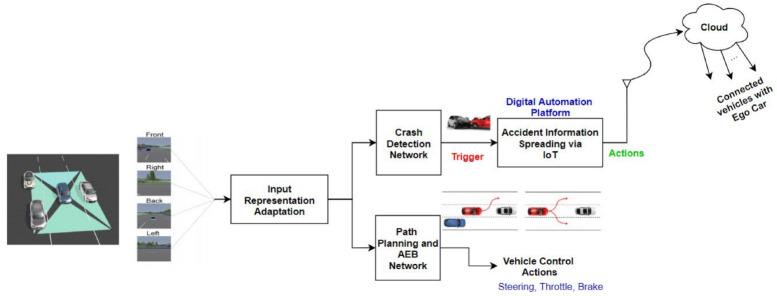
System architecture.

In this section, we tackle the proposed system architecture shown in Figure 1 in detail, and then we will describe the proposed neural networks models.

### 3.1. Input Representations

**Figure 2:**
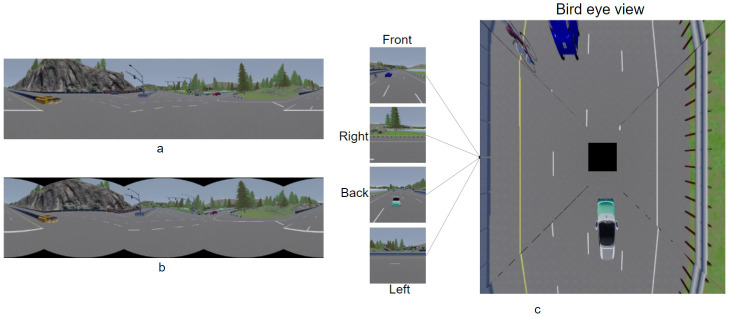
(**a**–**c**) Different input representation.

The proposed system depends on a camera cocoon setup that covers 360${}^{\circ }$ around the ego vehicle in which the input images can be adapted to different input representations. Input representations are shown in Figure 2 and can be front image only, normal stitching, equirectanguler, and bird’s eye view (BEV) warped images.

#### 3.1.1. Panorama—Normal Stitching

**Figure 3:**
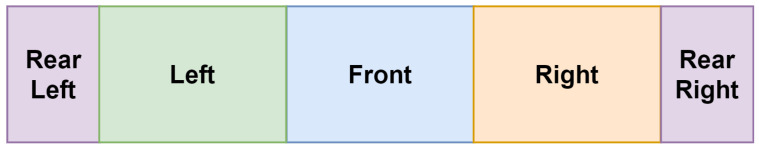
Normal stitching technique.

**Figure 4:**
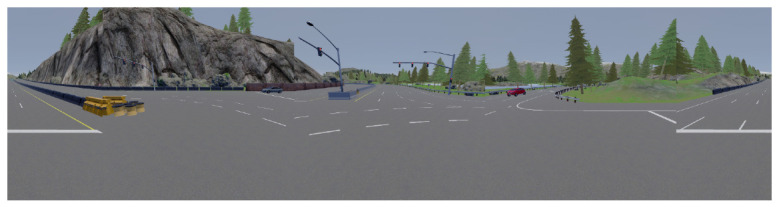
Normal stitching example.

Normal stitching panorama is achieved following the method shown in Figure 3 in which the front image will be in the middle, the left image is stitched to the left, and the right image is stitched to the right; however, the rear image is split into two subimages, where the right image is stitched to the right and the rear left image is stitched to the left. Figure 4 is an example of applying the normal stitching on the CARLA simulator. However, this view has a great drawback because the 4 images are not differentiated from each other, so it may fool the network when doing feature extraction.

#### 3.1.2. Panorama—Equirectangular Stitching

**Figure 5:**
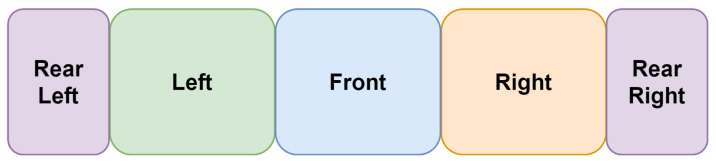
Panorama stitching technique.

**Figure 6:**
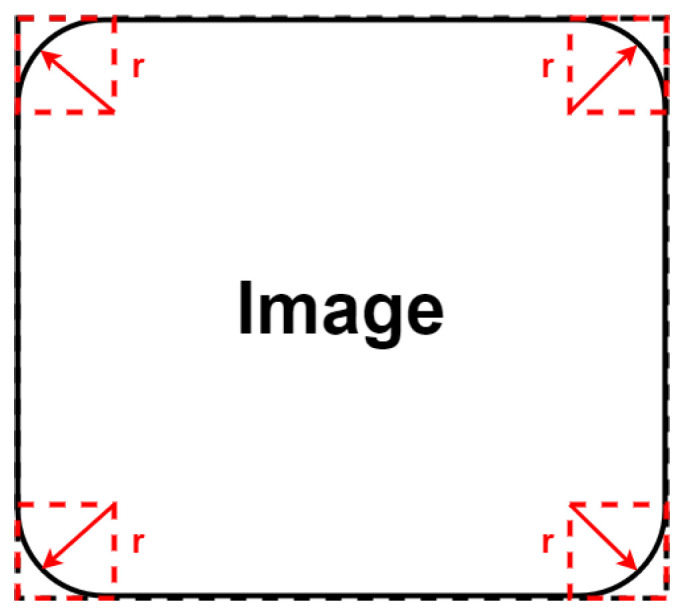
Tilting image with radius *r*.

**Figure 7:**
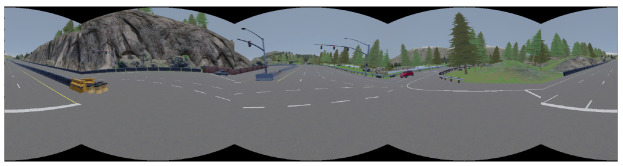
Panorama stitching example.

Equirectanguler stitching panorama is achieved following the same method of the normal stitching as shown in Figure 5. This stitching method solved the drawback of the normal stitching method in tilting the images from their edges (based on a tuned radius *r*) in order to differentiate between them, as shown in Figure 6. Figure 7 is an example of applying the normal stitching in the CARLA simulator. This example is simpler for use with the neural network than the normal stitching for doing features extraction and for differentiating between the 4 images.

#### 3.1.3. Bird’s Eye View (BEV)

Bird’s eye view is one of the most famous and trending types of view that is currently used in automotive applications. It depends on the camera calibration parameters. Intrinsic and extrinsic parameters warp the images taken from the bird’s eye view. Intrinsic parametersallow for mapping between the pixel coordinates and camera coordinates in the image frame, for example: optical center, focal length, and radial distortion coefficients of the lens when using a fisheye camera. Extrinsic parameters describe the orientation and location of the camera, where rotation and translation of the camera correspond to a world coordinate system.

**Figure 8:**
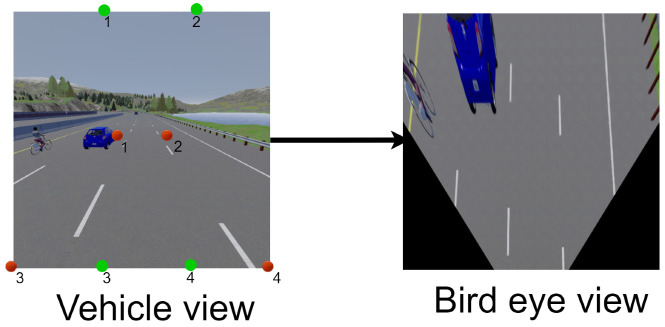
BEV transformation.

The warping algorithm is an image-transformation algorithm that depends on 8 predefined points: 4 points for the current view of the image and 4 points for the target transformed view. Figure 8 contains a vehicle view that is the normal front camera from the CARLA simulator with 4 red points (1,2,3,4) and 4 green points (1,2,3,4). The red points, and the trapezoidal shape, represent the vehicle view (current view), and the green points, and the rectangular shape, represent the target view (the image taken from this view). So, the main of warping algorithm is to transform these red points to green ones as shown in the bird’s eye view in Figure 8. Following the same procedure of warping to be applied on the left, right, and rear images as well, the full shape of the BEV is generated, but the question that remains to be answer is:what is this black square in the middle of the image? In fact, it is the ego-vehicle itself. After warping the 4 images part of the ego-vehicle appears, so it was necessary to remove it from the image in order to avoid fooling the neural network (which could lead to the vehicle taking incorrect actions, for example).

### 3.2. Multitask Neural Network

**Figure 9:**
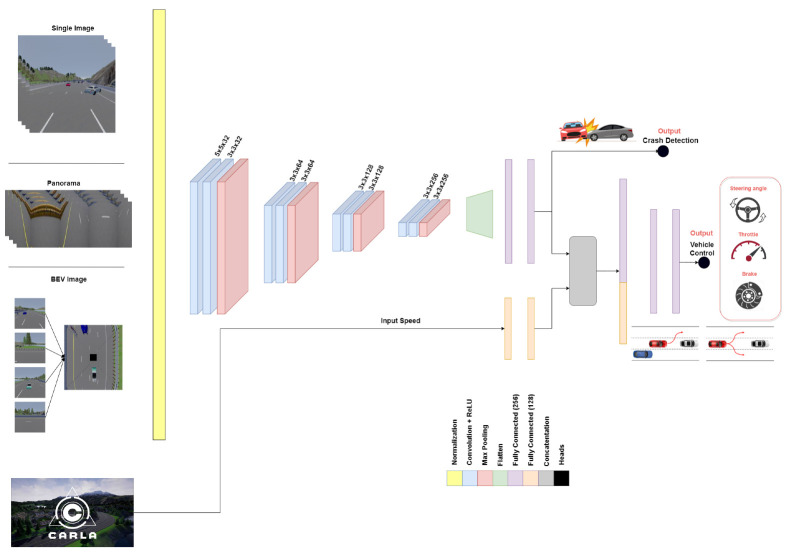
SDC-Net: multitask deep neural network.

The proposed system Figure 9 takes one of the input representations to SDC-Net, a multitask deep neural network that aims to perform two tasks together, as shown in Figure 1. Multitask network is defined as two output heads with different loss functions for each task; however, they are common in the same input representation and feature extraction part (backbone, neck, etc). First, the upper block is the crash detection network where a binary output is expected (i.e., 0 means no crash and 1 means crash). Thus, the second lower block is path planning and the AEB network that outputs the vehicle’s continuous control actions (i.e., steer, throttle, and brake).

Multitask deep neural network, inspired by [48] with some modifications, starts with an input normalization phase to achieve a zero mean and one standard deviation. Then, eight convolution layers in which the first convolution layer has a (5,5) kernel size, then the next seven convolution layers have a (3,3) kernel size, and the number of filters applied are 32,64,128, and 256, respectively, for every two consecutive convolution layers. Every convolution layer is followed by relu activation function introducing nonlinearties, and every two convolution layers are followed by a max pooling layer with a (2,2) kernel size. The output from the eighth convolution layer is flattened with dropout, and then two fully connected layers each have 256 neurons applied. The output of the second fully connected layer is divided into two paths. The first upper path is responsible for a crash detection network that aims to classify the scenes if there is a crash or when not using the binary cross entropy loss function. However, the second lower path passes through a concatenation layer with the feature extraction (two fully connected layers with 128 neurons) for the input speed, and then passes through another two fully connected layers with 256 neurons each. This second output aims to output the vehicle control actions by using a regression mean square error loss function. The output heads consist of downsized fully connected layers with dropouts where the classification path ended with a softmax layer to output the probabilities, and the regression path ended with a sigmoid layer to output the normalized continuous values for throttling, braking, and steering between 0 and 1. Table 1 shows each layer with the output shape, the number of parameters per layer, and the total number of parameters.

The normalization phase is considered to be a preprocessing phase applied on CARLA input images to ensure they have the same distribution. Eight convolutions are used as the starting point with a relatively large kernel size (5,5) to initially incorporate more information with a large receptive field. Then, finer kernel sizes of (3,3) are used to avoid the overfitting problem of using too many weights. The proposed network follows the rule of thumb "deeper is better than wider", because deeper networks learn more interesting features such as super features of the previous layer’s features. Thus, the number of filters applied is increased to encode the extracted features in the depth information while going deeper. Maxpooling layers are used to downsize the spatial features focusing on the most important features only. The second part of the network, especially the concatenation and fully connected layers, occur after the flattened layer that aims to mix the extracted features with CARLA signals that connect all the layers, and then prepare the network for the multiheads.

### 3.3. IoT Automation Platform

**Figure 10:**
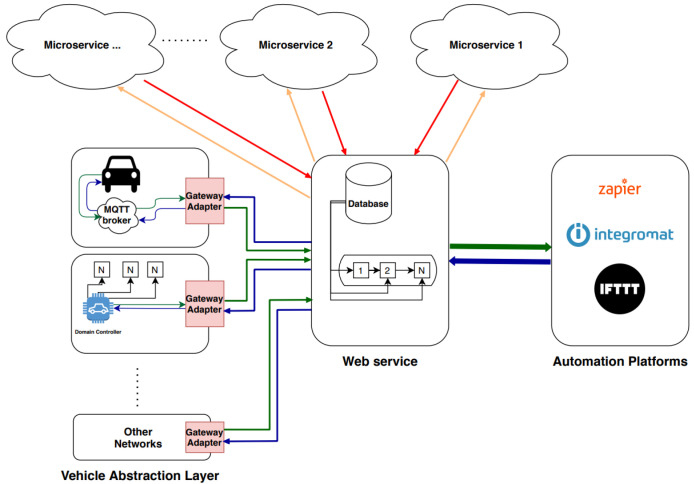
Digital gate system [49].

IoT digital automation platform is a trigger-action-based platform, if the trigger occurs, the IoT platform directly takes predefined actions using placeholder information. Reflecting this to our proposed system, the IoT platform aims to take the output from the crash detection network. Thus, if a crash is detected (trigger), then the IoT platform will act accordingly to provide the connected vehicles and traffic emergency patrols with relevant information about the accident (action). The shared information will be: the location of the accident, severity, and whether other vehicles are able to perform path planning to avoid accidents or must change their route. Otherwise, in case of no crash, there will not be a trigger, so no action is taken. The proposed system is integrated with the digital gate platform [45]—let’s do platform (LDO) [49] proposed by Valeo, which is considered an automotive standardization platform as shown in Figure 10.

Digital gate system is a system responsible for propagating data from vehicle communication buses (CAN, LIN, etc.) to automation platforms or microservices. Automation platforms are used for implementing trigger-action workflows. In addition, separate microservices are used to perform some of the logic needed for prototyping and proof of concept (POC) applications. A digital gate system consists of four main components, as presented in Figure 10. Vehicle abstraction layer is the first component in the system’s pipeline; it adapts data extraction from a vehicle and delivers this data to the gateway adapter library and also from the gateway adapter library to the vehicle. Gateway adapter library is responsible for communicating data between the vehicle abstraction layer and a web service. Moreover, the web service takes the role of routing the data, whether directly to the automation platforms or to the microservices, in order to process this data. Afterwards, the processed data goes to automation platforms or back to the vehicle. Furthermore, web services can interact with automation platforms (such as Zapier, IFTTT, Integromat, etc.) to perform user-customized work flows.

By projecting the previously described system into our proposed system, the multitask deep neural network is deployed onto one of the microservices. The camera cocoon images are sent from the vehicle abstraction layer to the web service via the gateway adapter. Accordingly, the web service routes the cocoon images to the SDC-Net microservice. SDC-Net adapts the cocoon to the best input representation (i.e., BEV, as we will see in the experimental results Section 5). Then, it sends information to the outputs of crash classification output (0 or 1) and vehicle continuous control actions (Throttle,steer,brake) ranges between 0 and 1 throughout the multiheads outputs. A postprocessing phase is performed to denormalize the control actions, then the web service receives these control actions and crash/no crash decision from SDC-Net microservice. Control actions are routed back to the vehicle abstraction layer via the gateway adapter where the vehicle controller applies these actions. However, crash/no crash decision is routed to the IoT automation platforms, where a previously created zap, applet, or scenario in Zapier, IFTTT, or Integromat, respectively. This zap/applet/scenario is a trigger-action-based workflow, so that if the trigger happens, the action is immediately taken. In case a crash decision is received, the workflow fires automatically to perform some specific tasks such as informing the connected vehicles of the location of a crash, its severity, and more. However, in case of a nocrash decision, the workflow will not fire and actions are taken.

## 4. Dataset Setup

Our proposed system is based on a camera cocoon setup using front, left, right, and rear cameras as shown in the system input in Figure 1 with different input representations as shown in Figure 2. As the proposed neural network is based on multitask learning, the cocoon cameras have two types of groundtruths, classification and regression labels. The classification label for crash detection is used with a binary label where zero:no crash detected and one:crash detected; however, the regression label for path planning and automatic emergency braking is used with continuous vehicle actions: throttle, steer, and brake. Due to lack of benchmarks that serve our proposed idea, we built our own dataset based on the CARLA simulator [33] covering difficult and various scenarios or situations that the vehicle may expose.

**Figure 11:**
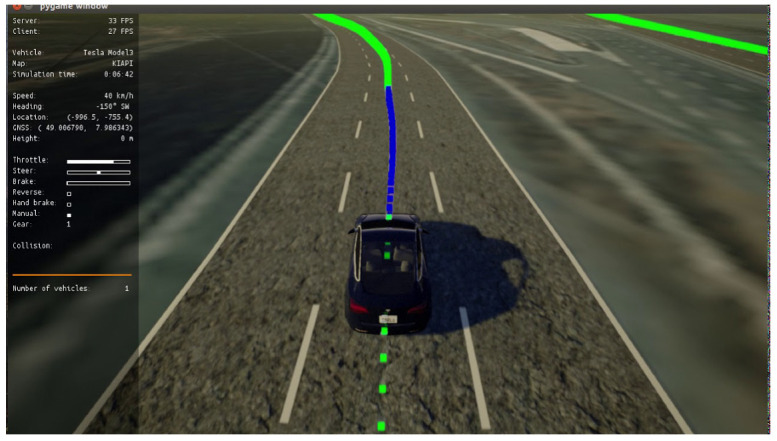
Carla simulator waypoints.

The CARLA dataset collection phase is composed of three main phases: waypoints generator, model predictive control (MPC), and scenarios saver. In **the first phase**, the CARLA simulator already generates waypoints (green points in Figure 11) based on the predefined maps (towns). A postprocessing is applied on these waypoints to act as the traditional baseline for path planning functionality. In **the second phase**, a model predictive control (MPC) [50] is used to control throttle, steer, and brake; it depends on a simple kinematic model to model the ego-vehicle which is a simplification for dynamic models ignoring tire forces, gravity, and mass since we are already using a simulator. MPC considers the task of following a trajectory as an optimization problem where the solution is the path the car should take. The idea is to simulate different actuator inputs (steer, throttle, and brake) and predict a resulting trajectory by selecting the one with the minimum cost. The car follows that trajectory and obtains a new input to calculate a new set of trajectories in order to optimize its path (blue points in Figure 11). The model utilizes a “horizon controller” which performs a trajectory recalculation for every new state, since the defined trajectory is just an approximation. The **state vector** is represented by [x,y,ψ,ν,cte,eψ] where: (x,y) is the ego-vehicle position, ψ is the ego-vehicle orientation, ν is the ego-vehicle velocity, cte is cross track error that is the difference between CARLA trajectory waypoints and current vehicle position in *y* coordinate, and eψ is the orientation error. For simplicity, brake and throttle are merged into a single actuation parameter (a), in addition to the steering actuation parameter (δ). MPC requires **actuator constraints**. This is why we put two constraints:

(1) a∈[−1,1]

(2) $$\delta \in [-25^{\circ },25^{\circ }]$$

The trajectory parameters are the number of time steps, *N*, separated in time by dt. Values for *N* and dt are chosen as 10 and 0.05 which causes the controller to predict 10 steps with a 0.5 section (500 ms) total duration. These values are chosen based on trial and error in order to achieve improved vehicle motion performance, where various combinations of *N* and dt produced erratic behavior due to the heavy processing that is needed. It is not necessary to use a large number of steps because the algorithm recalculates the trajectory for every step. Additionally, the large *N* is more costly to compute and causes the car to go off its path (especially > 20). The same occurs for dt. Smaller time steps are more costly, but larger values mean a lot of things happen between each calculation. The larger values cause the car run off track (especially 0.1). Accordingly, the controller predicts the trajectory of the vehicle during the preceding 0.5 s in the future. A 0.5 s prediction is sufficient for town 4, where the training data is collected, and 10 steps gives the balance between a discrete prediction and a reasonable processing time. The vehicle model state vector can be calculated from Equations (3)–(5), (7), and (8).

(3) $$x_{t+1}=x_{t}+\nu_{t}*cos\left(\psi_{t}\right)*dt$$

(4) $$y_{t+1}=y_{t}+\nu_{t}*sin\left(\psi_{t}\right)*dt$$

(5) $$\psi_{t+1}=\psi_{t}+\frac{\nu_{t}}{L_{f}}*\delta_{t}*dt$$

(6) $$\nu_{t+1}=\nu_{t}+a_{t}*dt$$

(7) $$cte_{t+1}=f\left(x_{t}\right)-y_{t}+\nu_{t}*sin\left(e\psi_{t}\right)*dt$$

(8) $$e\psi_{t+1}=\psi_{t}-{\psi_{des}}_{t}+\frac{\nu_{t}}{L_{f}}*\delta_{t}*dt$$

where $L_{f}$ is the radius formed by running the vehicle in the simulator around in a circle with a constant steering angle and velocity on a flat terrain, its value equals (2.9–3.0) in the CARLA simulator town. $f(x_{t})$ is the substitution of $x_{t}$ in the polynomial function of waypoints generated by CARLA. The loss function aims to minimize the difference between the trajectory created by the CARLA simulator and the vehicle’s current position (cte). Additionally, the orientation error (eψ) can be seen across *N* time steps, as shown in Figure 9.

(9) $$J=\sum_{t=1}^{N}{\left(cte_{t}-cte_{ref}\right)}^{2}+{\left(e\psi_{t}-e\psi_{ref}\right)}^{2}$$

Our benchmark dataset is collected using around 500 episodes that contain 125 K dataframes: 380 dataframes with 95 K samples; 80 K samples for training; and 15 K samples for validation using town 4 only. However, there are 120 dataframes with 30 K samples for testing using town 5. Table 2 shows that the training data is split into 35 K crash samples and 45 K no crash samples. Validation data is also split into 6 K crash samples and 9 K no crash samples. The testing data is split equally into 15 K crash and no crash samples each. The CARLA autopilot functionality is used to augment our collected dataset especially for no crash samples depending on the simple rule-based path planning provided by CARLA. In **the third phase**, each dataframe saves front, left, right, and rear camera images in addition to the vehicle control actions of throttle, brake, and steer. Each episode starts from a random location from predefined locations set on the map and finishes when the vehicle reaches the destination. The number of vehicles, its types, and colors are also tuned. Our data is collected to cover many various and different scenarios including when the ego-vehicle is moving and other vehicles are static and when the ego-vehicle and other vehicles are dynamic in order to ensure we had a robust dataset. Training, validation, and testing data cover many scenarios; some of these scenarios are as follows:

- Front crash when the ego-vehicle moves to right most, left most, and middle lanes;
- Left crash when the ego-vehicle moves the same and to a right lane;
- Right crash when the ego-vehicle moves the same and to a left lane;
- Front vehicle moves with lower speed to check lane overtaking;
- Two static front adjacent vehicles block the ego-vehicle;
- Two dynamic front adjacent vehicles move at the same velocity;
- Two dynamic front adjacent vehicles move at different velocities;
- Left vehicle moves beside the ego-vehicle at the same velocity;
- Ego-vehicle crashes with a front vehicle (achieved by using large number of time steps *N* in MPC), etc.

**Table 2:** Number of Samples.

	Training Data	Validation Data	Testing Data
**Crashes**	35 K	6 K	15 K
**No Crashes**	45 K	9 K	15 K

**Figure 12:**
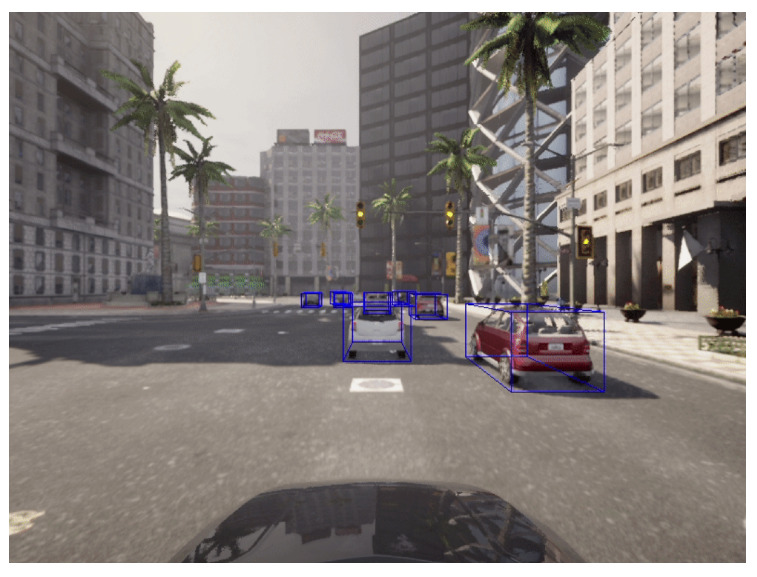
Carla example with projected bounding boxes.

**Figure 13:**
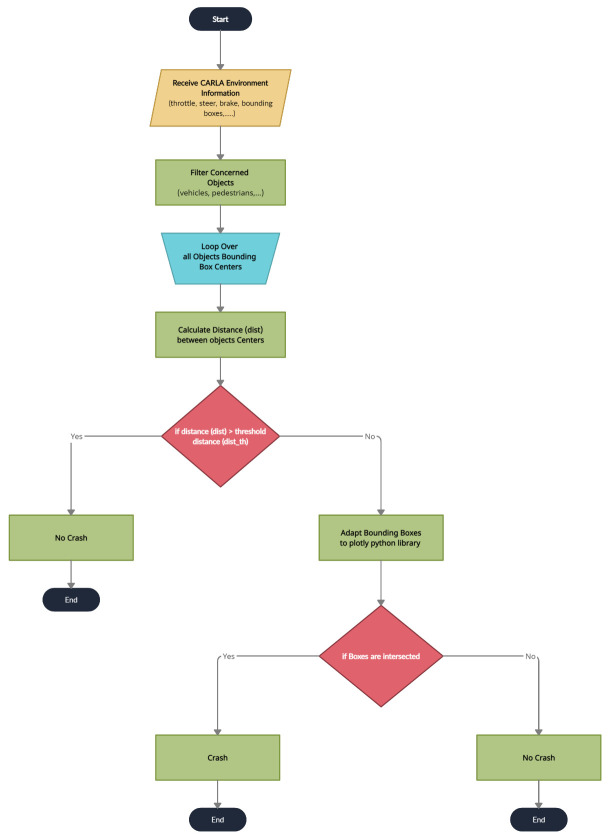
Crash labels processing flowchart.

The CARLA simulator provides the ego-vehicle information, (speed, steer, and brake), that can be saved during the data collection phase. The simulator also provides built-in **sensor fusion** algorithms that are able to localize and track other vehicles, so the ego-vehicle knows all of the relevant environmental information. One of these datapoints is the position of each vehicle that can be represented as bounding boxes such as center, length, width, and height data with respect to the global coordinates as shown in Figure 12. Although the CARLA simulator has collision information, our proposed system also generates crash labels on a processing phase as shown in the flowchart in Figure 13 and it is composed of the following steps:

- Filter the objects by keeping only the concerned objects such as vehicles, pedestrians, etc.
- Loop over all the bounding boxes centers received from the CARLA simulator;
- Calculate the distances between bounding boxes centers;
- Check if the distances are greater than the threshold tunable distance. If yes, no crash label is applied; however, if no, this means that we have two or more vehicle centers in close proximity to each other;
- Adapt the bounding boxes information to the plotly [51] python library to check if there are two intersecting boxes;
- Check if the boxes are intersecting. If yes, apply the crash label. If no, apply the no crash label.

For the path planning and AEB, safety aspects are also taken into consideration where the ego-vehicle will not be able to perform lane overtaking if and only if one of the adjacent lanes is empty, otherwise the ego-vehicle will perform AEB. Lane overtaking is performed if the waypoints shrink and the CARLA simulator detects that there is an object in front of the ego-vehicle. Traffic lights are also respected, especially in the augmented autopilot data. This means that is is difficult for the neural network to learn the differentiation between AEB and stops that are respecting traffic lights.

## 5. Results

Experiments were conducted based on two variants: different input representation and one/multi head outputs. The first variant used was front camera only, panorama (stitched), and bird’s eye view (BEV); however, the second variant used was the proposed system with one output head: crash detection only (classification), control actions only (regression), and then both output heads together. The main aim behind the second variant of experiments was to test the multitask effectiveness. The most common measurement metrics (KPIs) that we depended on for our results for the classification problem are accuracy, precision, recall, and F1-score, while for the regression problem we used the mean square error (MSE).

Based on the previously mentioned variants, our experimental results are shown in Table 3. We show the different input representation in rows and different experiments in columns. For each experiment, the relevant metrics are reported for the testing data (i.e., precision, recall, F1-score, accuracy for classification, and MSE for regression). We categorized experimental results by experiments, then within each experiment. Finally, we compared between the different input representations.

### 5.1. Crash Avoidance Only Results

The crash avoidance only experiment is considered a single-head classification output network (not multitask) where the experiment is conducted three times: one using only the front camera, one using only the panorama view, and the last one using only the BEV. Due to the classification problem, the measurement metrics are precision, recall, f1-score, and accuracy. It is obvious that BEV results are better than the panorama camera and front camera results in precision by 8.88% and 15.93%, respectively; in recall by 6.75% and 19.11%, respectively; in f1-score by 7.77% and 17.7%, respectively; and in accuracy by 4% and 11%, respectively.

### 5.2. Path Planning and AEB Only Results

The path planning and AEB only experiment is considered to be another single-head experiment but with a regression output network (not multitask) where the experiment is also conducted three times: one using only the front camera, one using only the panorama view, and the last one using only BEV. Due to the regression problem, the measurement metric is MSE for the control actions (throttle, steer, and brake). It is obvious that the BEV results are better than the panorama camera and front camera results in throttle MSE by 8.64% and 10.2%, respectively; in steer MSE by 2.314% and 10.7%, respectively; in brake MSE by 0.3932% and 14.946%, respectively.

### 5.3. Crash Avoidance, Path Planning, and AEB Results

Crash avoidance, path planning, and AEB experiments are considered to be a multihead classification–regression output networks (multitask) where the experiment is conducted three times: one using only the front camera, one using only the panorama view, and the last one using only using the BEV. Due to the multitask problem, the measurement metrics are the already previously mentioned ones of precision, recall, f1-score, and accuracy, in addition to the control actions MSE. It is obvious that the BEV results are better than panorama camera and front camera results in precision by 11.62% and 23.58%, respectively; in recall by 9.43% and 28.58%, respectively; in f1-score by 10.53% and 26.22%, respectively; in accuracy by 6% and 13%, respectively; in throttle MSE by 26.86% and 41.28%, respectively; in steer MSE by 23.55% and 43.16%, respectively; and in brake MSE by 27.11% and 46.026% respectively.

As a **discussion for the crash avoidance functionality**, the experiments using only the front camera succeeded in detecting crashes that occurred in the FOV of the camera in front of the ego-vehicle only; however, it failed in detecting crashes beside or behind the ego-vehicle. This is because the ego-vehicle was not visually seeing these crashes, so the measurement metrics for front camera experiments are not so good, which was already expected. However, the panorama and BEV experimental setups depended on a cocoon camera covering 360${}^{\circ }$ around the ego vehicle, so both of them had better measurement metrics, but **why did the BEV experiments outperform the panorama experiments?** BEV depends on warping images where distance features are extracted easily by the neural network detecting if the vehicles collide with each other or not; however, the panorama input representation depends on stitching images, taken directly from the cameras, together as mentioned previously, so the neural networks may not be able to estimate the distances between vehicles (collision detection).

As a **discussion for path planning and AEB functionalities**, the experiments using only the front camera succeeded in performing AEB very well because it depended only on the front scene; however, regarding path planning, the experiment with only the front camera failed in scenarios with other vehicles beside the ego-vehicle. This is because there is a lack of information about the sides and rear of the vehicle, so the front camera only method is not safe for path planning. However, panorama and BEV experiments fill the gap in the information by using a camera cocoon setup, so both of them provided better measurement metrics compared with using only the front camera. Again, we must ask **why the BEV experiments outperformed the panorama experiments?** The panorama input representation depended on stitching images taken directly from the cameras together, as mentioned previously, so the neural network is not always able to differentiate between front, left, right, and rear cameras while performing path planning. On the other hand, BEV depends on warping images where the front camera image is located in the upper part of the warped/projected image, the left camera image is located in the left part of the warped/projected image, the right camera image is located in the right part of the warped/projected image, and the rear camera image is located in the lower part of the warped/projected image. The warping or projection helps the neural network to **extract the surrounding features** effectively and then conduct the path planning.

**Figure 14:**
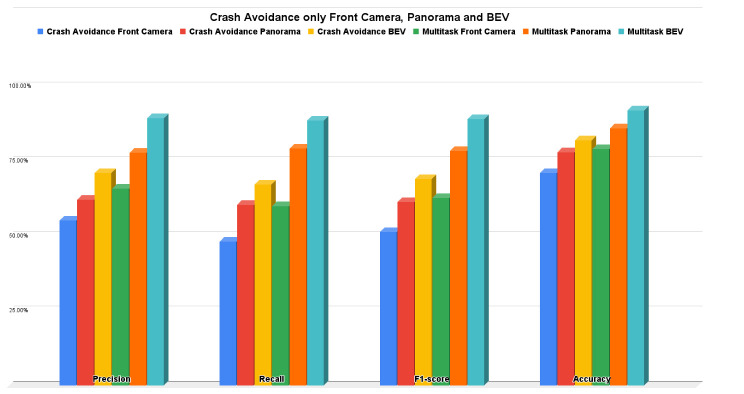
Precision, recall, f1-score, and accuracy for crash avoidance only vs. multitask.

**Figure 15:**
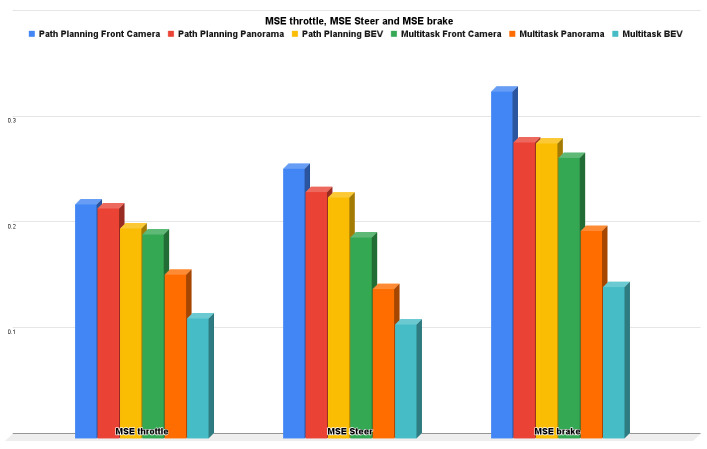
MSE throttle, steer, and brake for path planning for AEB only vs. multitask.

Conceptually, multitask networks perform very well and also perform better than single-purpose networks because both tasks help each other to achieve improved performance. Figure 14 shows the bar charts for precision, recall, f1-score, and accuracy for all the experiments we conducted; multitask with the BEV input bar is the best higher one in the whole metric. Figure 15 shows the bar charts for the MSE of throttle, brake, and steer for the all the conducted experiments; multitask with BEV input bar is also the best lower one in the whole metric. Overall, the multitask experiments are better than other experiments. The front camera experiments proved that when we neglect some environmental information (left, right, and rear images), the results are not good when compared with panorama and BEV input representations. The panorama results are good; however, BEV is the best thanks to the warping/projection that gives additional information to the neural network, especially for crash avoidance and path planning.

## 6. Conclusions

The dream of a self-driving car can be achieved through the collaboration between sensor fusion, deep learnin, and IoT. Crash avoidance, path planning, and automatic emergency braking are essential to achieve an autonomous driving system. As a result, we proposed SDC-Net: an end-to-end multitask system based on camera cocoon and IoT. Our system is able to automatically control vehicles to accelerate, maneuver, and brake in addition to detecting crashes and sharing the crash information with all connected vehicles. Our benchmark dataset based on the CARLA simulator was built to cover difficult and varied scenarios because of the lack of benchmarks serving our system. Extensive experiments were conducted using different input representations, and the experiments proved that the multitask neural network with a BEV input outperformed the other methods.

## Figures and Tables

**Table 1:** Multitask network parameters.

Layer	Output Shape	Number of Parameters
Input Layer	(None, 120, 300, 3)	-
Conv1	(None, 120, 300, 32)	2432
Conv2	(None, 120, 300, 32)	9248
MaxPool1	(None, 60, 150, 32)	-
Conv3	(None, 60, 150, 64)	18,496
Conv4	(None, 60, 150, 64)	36,928
MaxPool2	(None, 30, 75, 64)	-
Conv5	(None, 30, 75, 128)	73,856
Conv6	(None, 30, 75, 128)	147,584
MaxPool3	(None, 15, 38, 128)	-
Conv7	(None, 15, 38, 256)	295,168
Conv8	(None, 15, 38, 256)	590,080
MaxPool4	(None, 8, 19, 256)	-
Flatten	(None, 38,912)	-
FC1-256	(None, 256)	9,961,728
FC2-256	(None, 256)	65,792
Input Speed	(None, 1)	-
FC1-128	(None, 128)	256
FC2-128	(None, 128)	16,512
Concat (FC2-256, FC2-128)	(None, 384)	-
FC3-256	(None, 256)	98,560
FC4-256	(None, 256)	65,792
Crash Head (FC1-10)	(None, 10)	2570
Crash Head (FC2-1)	(None, 1)	11
Softmax	(None, 1)	-
Control Head (FC1-10)	(None, 10)	2570
Control Head (FC2-3)	(None, 3)	33
Sigmoid	(None, 3)	-
		11,387,616

**Table 3:** Measurement metrics comparison.

		Experiments													
		Crash Avoidance Only				Path Planning and AEB Only			Crash Avoidance, Path Planning and AEB						
**Input** **Representations**		Precision	Recall	F1-Score	Accuracy	MSE throttle	MSE Steer	MSE brake	Precision	Recall	F1-Score	Accuracy	MSE throttle	MSE Steer	MSE brake
	Front Camera	0.5513	0.4804	0.5134	0.71	0.2214	0.2552	0.3285	0.6589	0.6	0.628	0.79	0.1933	0.1902	0.2655
	Panorama	0.6218	0.604	0.6127	0.78	0.2176	0.2333	0.2805	0.7785	0.7915	0.7849	0.86	0.1552	0.1414	0.1966
	Bird Eye View (BEV)	0.7106	0.6715	0.6904	0.82	0.1988	0.2279	0.2794	**0.8947**	**0.8858**	**0.8902**	**0.92**	**0.1135**	**0.1081**	**0.1433**

## Data Availability

Not applicable.
